# CCL19/CCR7 drives regulatory T cell migration and indicates poor prognosis in gastric cancer

**DOI:** 10.1186/s12885-023-10882-7

**Published:** 2023-05-19

**Authors:** Danhua Xu, Xu Liu, Shouyu Ke, Yixian Guo, Chunchao Zhu, Hui Cao

**Affiliations:** Department of Gastrointestinal Surgery, Ren Ji Hospital, School of Medicine, Shanghai Jiao Tong University, Shanghai, China

**Keywords:** Chemokines, CCL19, CCR7, Treg cells, CD8 + T cells, Gastric cancer

## Abstract

**Background:**

Gastric cancer is associated with significant morbidity and mortality in the world. Blocking programmed cell death protein 1 pathway have been approved for the treatment of a variety of tumors and have achieved remarkable clinical therapeutic effects. However, immune checkpoint inhibitors failed to achieve satisfactory results in gastric cancer. There is a need to identify novel immunotherapy targets in gastric cancer.

**Methods:**

We analysed the correlation between Treg cells and CD8 + T cells in gastric cancer samples. We studied the relationship between chemokines and Treg cells or CD8 + T cells in gastric cancer. We compared CCL19/CCR7 expression in gastric cancer patients in TCGA database. We performed transwell experiments to determine the influence of CCL19 on Treg cells and CD8 + T cells migratory capacity. We conducted survival analysis of CCL19 and CCR7 in gastric cancer database.

**Results:**

Treg cells show positive correlation with CD8 + T cells in gastric cancer. Treg cell expression was significantly upregulated in tumor tissues. Patients with high FOXP3 expression had worse overall survival than those with low FOXP3 expression. CCL19 had strong correlation with FOXP3 and weak correlation with CD8A. CCL19 had strong impact on the migratory capacity of Treg cells but weak impact on the migratory capacity of CD8 + T cells. Both CCL19 and CCR7 expression were significantly upregulated in gastric cancer tissues. Survival analysis demonstrated that both CCL19 and CCR7 indicate poor prognosis in gastric cancer.

**Conclusions:**

CCL19/CCR7 may be a potential novel therapeutic target in gastric cancer.

## Background

Gastric cancer is associated with significant morbidity and mortality. In 2020, there were more than 1 million new cases of gastric cancer and more than 700 thousand deaths worldwide, being the fifth incidence rate of malignant tumor and the fourth leading cause of cancer-related deaths in the world [1]. Most patients present with advanced disease, thus leading to poor survival. Less than 15% of patients with metastasis live for more than 2 years [2]. Nowadays gastric cancer treatment still depends on classical surgery, chemotherapy, and targeted therapy. Systemic chemotherapy has been shown to improve survival when compared to supportive care; however, even with optimal chemotherapy, the median survival for gastric cancer patients treated in first line clinical trials is less than 1 year [3]. Trastuzumab and ramucirumab have resulted in modest improvements in overall survival for patients with HER2-positive gastric cancer [4, 5]. Pembrolizumab and nivolumab that block programmed cell death protein 1 (PD-1) pathway have been approved by Food and Drug Administration (FDA) for the treatment of a variety of tumors and have achieved remarkable clinical therapeutic effects [6]. Nevertheless, immune checkpoint inhibitors failed to achieve satisfactory results in gastric cancer and studies on more effective and selective immunotherapy drugs need to be undertaken [7].

Regulatory T cells (Treg cells) play an important role in maintaining self-tolerance and are enriched in many cancer types. Treg cells suppress effector T cells and natural killer cells by secreting soluble immunosuppressive factors such as transforming growth factor-β (TGF-β), and expressing inhibitory receptors such as cytotoxic T-lymphocyte antigen 4 (CTLA-4) [8, 9]. An increase of tumor infiltrating Treg cells has been correlated with poor prognosis in many cancer types [10–12]. Treg cells infiltrate into the tumor microenvironment (TME) and play a significant role in suppressing anti-tumor immune responses [12–15], making them an obstacle to effective cancer immunotherapy. Elimination of Treg cells with an anti-CD25-coupled toxin has shown satisfactory effect in early-phase clinical trials [16, 17]. The clinical benefit of checkpoint blockade with anti-CTLA4 has also been partially attributed to depletion of intratumoral Treg cells [18]. These results suggest that Treg cells are promising targets for anti-tumor immunotherapy. However, systemic suppression of Treg cells can induce severe autoimmune complications [19]. Cell movement is the essential biological process regulated by chemokines and their receptors. Chemokines can promote cell migration, or stimulate cell adhesion, causing cell movement to stop [20–22]. The migratory capacity of Treg cells is a critical factor impacting their ability to regulate tissue-restricted inflammation [23]. Targeting chemokines and chemokine receptors may therefore be an attractive approach to elicit beneficial anti-tumor immune responses in patients.

## Materials and methods

### Patients

In the study, we collected samples from 20 patients with resected primary gastric cancer who were treated at Ren Ji Hospital, School of Medicine, Shanghai Jiao Tong University. The diagnosis was confirmed by a review of the H&E stained slides. The inclusion criteria for the study were as follows: (1) an obvious pathologic diagnosis of gastric cancer; (2) primary gastric cancer cases without a history of other solid tumours; (3) no exposure to chemotherapy, radiotherapy, or other anti-cancer therapies before surgery.

### Flow cytometry

The following flow cytometry antibodies were purchased from Biolegend: CD3 (OKT3), CD8 (RPA-T8). FOXP3 (PCH101), CD25 (BC96) were from eBiosciences. Antibodies staining was performed according to the manufacturer’s instructions (eBioscience). Cells were stained in PBS containing 2% fetal bovine serum (FBS) with antibodies as indicated. All samples were processed on a LSRFortes-saTM X-20 flow cytometer (BD Biosciences) and data were analyzed by FlowJo software.

### Immunohistochemical staining

The paraffin-embedded tissue samples were used for immunohistochemical (IHC) staining. Briefly, after tissue sections were deparaffinised, rehydrated with graded ethanol, incubated with 0.3% hydrogen peroxide for 30 min, and blocked with 10% bovine serum albumin (BSA) (Sangon, Shanghai, China), slides were first incubated overnight with an antibody against FOXP3 (dilution 1:200, CST, USA, 12,653) at 4 °C, and then labelled with an HRP-conjugated (rabbit) secondary antibody (Thermo-Fisher Scientific, USA) at room temperature for 1 h. Finally, positive staining was visualized using diaminobenzidine (DAB) substrate liquid (Gene Tech, Shanghai), followed by counterstaining with haematoxylin. All sections were observed and photographed with a microscope (Carl Zeiss, Germany).

### Cell culture and reagents

Peripheral blood mononuclear cells (PBMCs) were isolated by density gradient centrifugation using Ficoll-Paque (GE Healthcare). Treg cells and CD8 + T cells were isolated from human peripheral blood by FACS on a BD FACS ARIA II sorter (BD Biosciences). Cells were stimulated under anti-CD3/anti-CD28 antibody for 48 h. Proliferative cells were further detected by flow cytometry.

Human gastric cancer cell line BGC-823 was maintained at Shanghai Cancer Institute, Ren Ji Hospital, School of Medicine, Shanghai Jiao Tong University. Cells were cultured according to American Type Culture Collection (ATCC, Manassas, VA) protocols, supplemented with 10% (v/v) fetal bovine serum (FBS) and 1% antibiotics at 37 °C in a humidified incubator under 5% CO2 conditions.

### Transwell experiment

The migratory capacity of Treg cells and CD8 + T cells was measured by a Transwell assay (Corning, NY, USA) according to the manufacturer’s instructions. About 2 × 10^4^ cells in 200 µl medium were seeded into the upper chamber of the Transwell inserts. RPMI 1640 medium containing 10% (v/v) FBS was added to the bottom chamber. Cells were incubated at 37 °C and allowed to migrate for 24 h. At the designated time points, the migrated cells were detected by flow cytometry.

### Immunofluorescence staining

Paraffin Sect. (5 mm) of human gastric cancer tissues were deparaffinized, rehydrated with graded ethanol. After blocking with 10% BSA, sections were incubated with primary antibodies for 1 h followed by incubation with secondary antibodies for 30 min at room temperature. For staining of CCL19, primary and secondary antibodies were anti-CCL19 (1:100, RD, USA, MAB361), anti-rabbit Alexa Fluor 594 (1:400, Jackson ImmunoResearch, #711–585 − 152). For staining of FOXP3, primary and secondary antibodies were anti-FOXP3 (1:400, CST, USA, 12,653), anti-rabbit Alexa Fluor 594 (1:400, Jackson ImmunoResearch, #711–585 − 152). Nuclei were counterstained with DAPI (40,6-diamidino-2-phenylindole dihydrochloride; AppliChem, A4099). Digital images were acquired with fluorescence or confocal microscopes equipped with a digital camera (Nikon).

### Bioinformatics analysis

The gene expression data for stomach adenocarcinoma (STAD) were downloaded from The Cancer Genome Atlas (TCGA) maintained by Broad Institute’s TCGA workgroup.

### Statistical analysis

Graphical representations were prepared with GraphPad Prism 7 (San Diego, CA, USA) software. The SPSS software program (version 19.0, IBM Corporation) was used for statistical analysis. Survival curves were evaluated using the Kaplan-Meier method and differences between survival curves were tested by the log-rank test. Data were presented as means ± SD. All statistical tests were two-sided. The Student’s t-test or one-way ANOVA was used for comparison between groups. P < 0.05 was considered statistically significant.

## Results

### Tumor infiltrating Treg cells show positive correlation with CD8 + T cells in gastric cancer

**Fig. 1:**
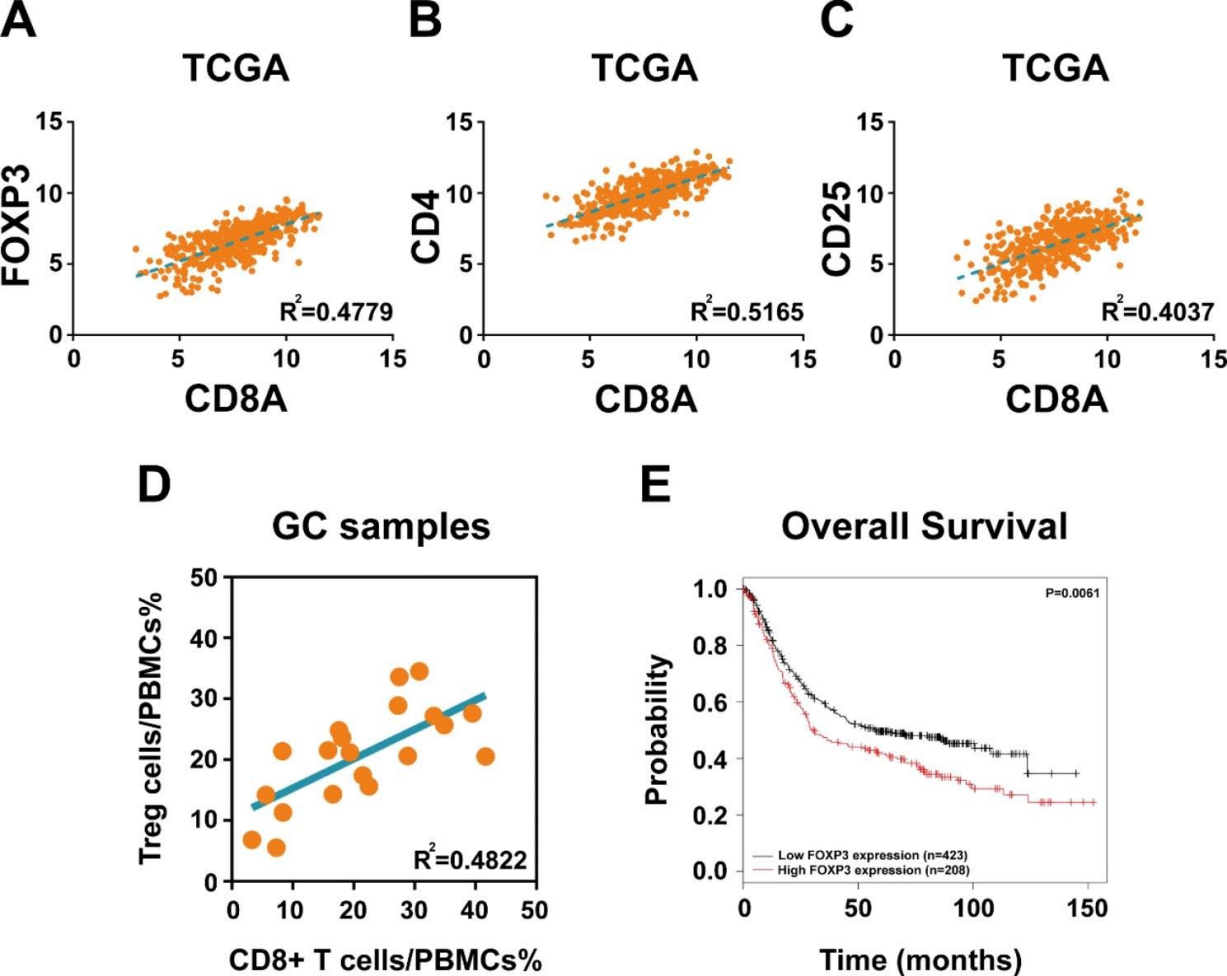
Treg cells show positive correlation with CD8 + T cells in gastric cancer. (**A**) Pearson’s correlation coefficient revealed a positive correlation between FOXP3 and CD8A expression in TCGA database. (**B**) Pearson’s correlation coefficient revealed a positive correlation between CD4 and CD8A expression in TCGA database. (**C**) Pearson’s correlation coefficient revealed a positive correlation between CD25 and CD8A expression in TCGA database. (**D**) Flow cytometry assay showed positive correlation between Treg cells and CD8 + T cells in 20 fresh gastric cancer samples. (**E**) KMplotter database demonstrated that patients with high FOXP3 expression had worse overall survival than those with low FOXP3 expression

The correlation analysis between Treg cell signature genes (CD4, CD25 and FOXP3) and CD8A in The Cancer Genome Atlas (TCGA) database containing 415 gastric cancer samples revealed that the expression of CD4, CD25 and FOXP3 was positively correlated with CD8A expression respectively (Fig. 1A C). To validated the results of bioinformatics analysis, 20 fresh clinical gastric cancer samples were collected. Flow cytometry assay also showed positive correlation between Treg cells and CD8 + T cells in gastric cancer tissues (Fig. 1D). Analysis of 631 gastric cancer patients with 33 months of follow-up data in KMplotter database demonstrated that patients with high FOXP3 expression had worse overall survival (OS) than those with low FOXP3 expression, suggesting that Tregs cells indicate poor prognosis in gastric cancer (Fig. 1E).

### Expression of Treg cells is elevated in gastric cancer tissues compared with that in normal tissues

**Fig. 2:**
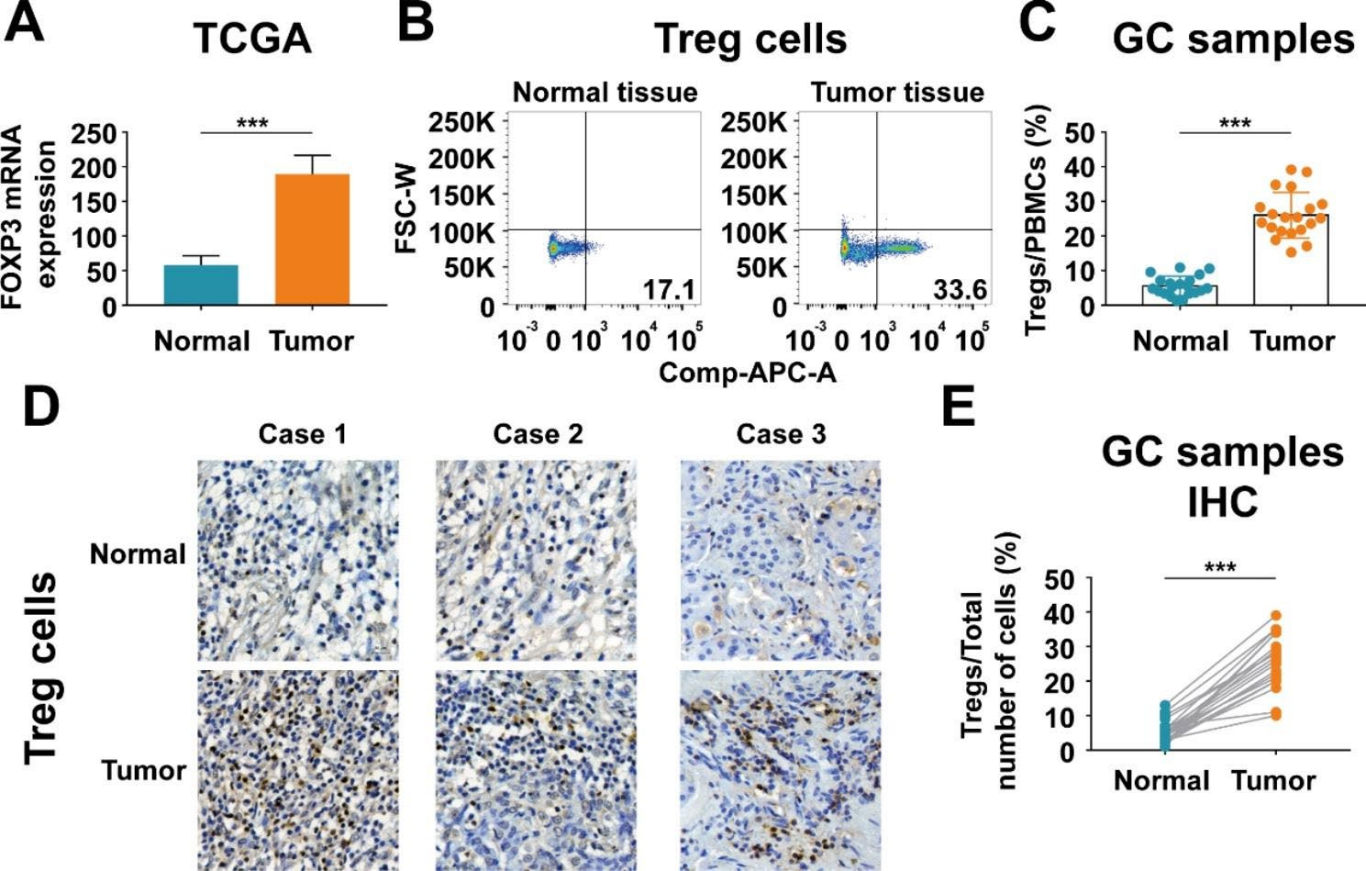
Expression of Treg cells is elevated in gastric cancer. (**A**) Relative FOXP3 mRNA expression in 32 pairs of cancerous and noncancerous tissues from gastric cancer patients in TCGA database. (**B-C**) Flow cytometry assay demonstrated elevated expression of Treg cells in fresh samples from 20 gastric cancer patients. (**D-E**) Immunohistochemistry staining showed that expression of Treg cells was significantly increased in gastric cancer tissues compared with that in normal tissues. Values are mean ± s.d., ***P < 0.001 (Student’s t-test)

Comparing gene expression in 32 pairs of cancerous and noncancerous tissues from gastric cancer patients in TCGA database revealed that FOXP3 expression was significantly upregulated in tumor samples compared with that in matched normal samples (Fig. 2A). To validated the results of database analysis, flow cytometry assay was performed using 20 fresh gastric cancer samples and their matched normal samples. The results demonstrated elevated expression of Treg cells in tumor tissues consistent with the bioinformatics analysis (Fig. 2B C). Immunohistochemistry staining also showed that expression of Treg cells was significantly increased in gastric cancer tissues compared with that in normal tissues (Fig. 2D and E).

### Chemokine CCL19 has strong correlation with FOXP3 and weak correlation with CD8A

**Fig. 3:**
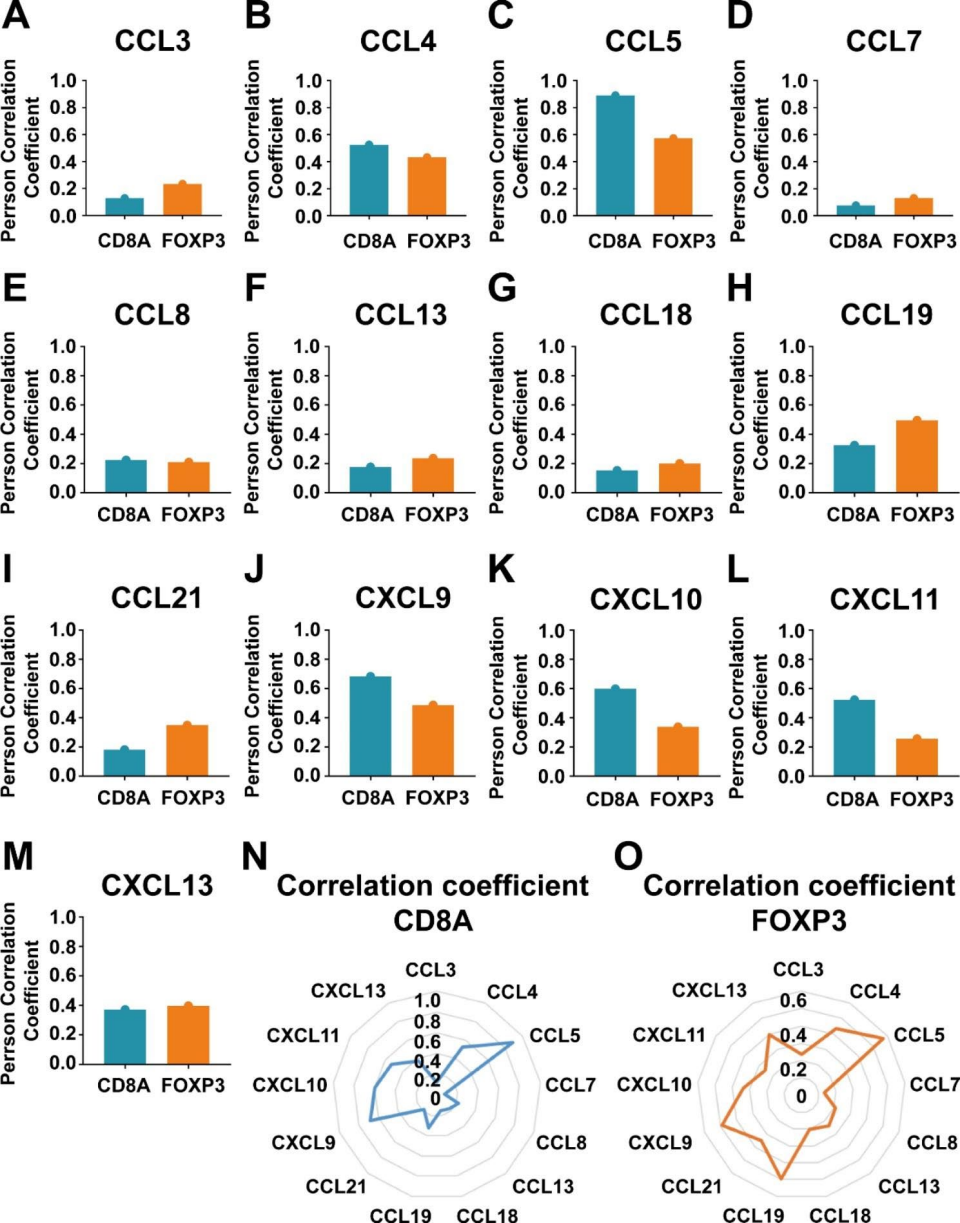
The correlation analysis among chemokines, FOXP3 and CD8A in TCGA database. (**A-M**) Pearson’s correlation coefficient between chemokines (CCL3, CCL4, CCL5, CCL7, CCL8, CCL13, CCL18, CCL19, CCL21, CXCL9, CXCL10, CXCL11, CXCL13) and FOXP3, CD8A in TCGA database. (**N-O**) Radar map showed the correlation coefficient between chemokines and FOXP3, CD8A in TCGA database

Then we used TCGA database to study the relationship among chemokines, Treg cells and CD8 + T cells in gastric cancer. The correlation analysis showed that chemokine CCL3, CCL7, CCL8, CCL13, CCL18, CCL21 or CXCL13 has both weak correlation with FOXP3 and CD8A (Fig. 3A, D, G and I M). Chemokine CCL4, CCL5 or CXCL9 has both strong correlation with FOXP3 and CD8A (Fig. 3B C, 3J). Chemokine CXCL10 or CXCL11 has weak correlation with FOXP3 and strong correlation with CD8A (Fig. 3K L). Only chemokine CCL19 has strong correlation with FOXP3 and weak correlation with CD8A (Fig. 3H N-3O), suggesting that CCL19 may be a potential target to inhibit Treg cell infiltration, but may not affect CD8 + T cell infiltration.

### Chemokine CCL19 has strong chemotactic activity on Treg cells

**Fig. 4:**
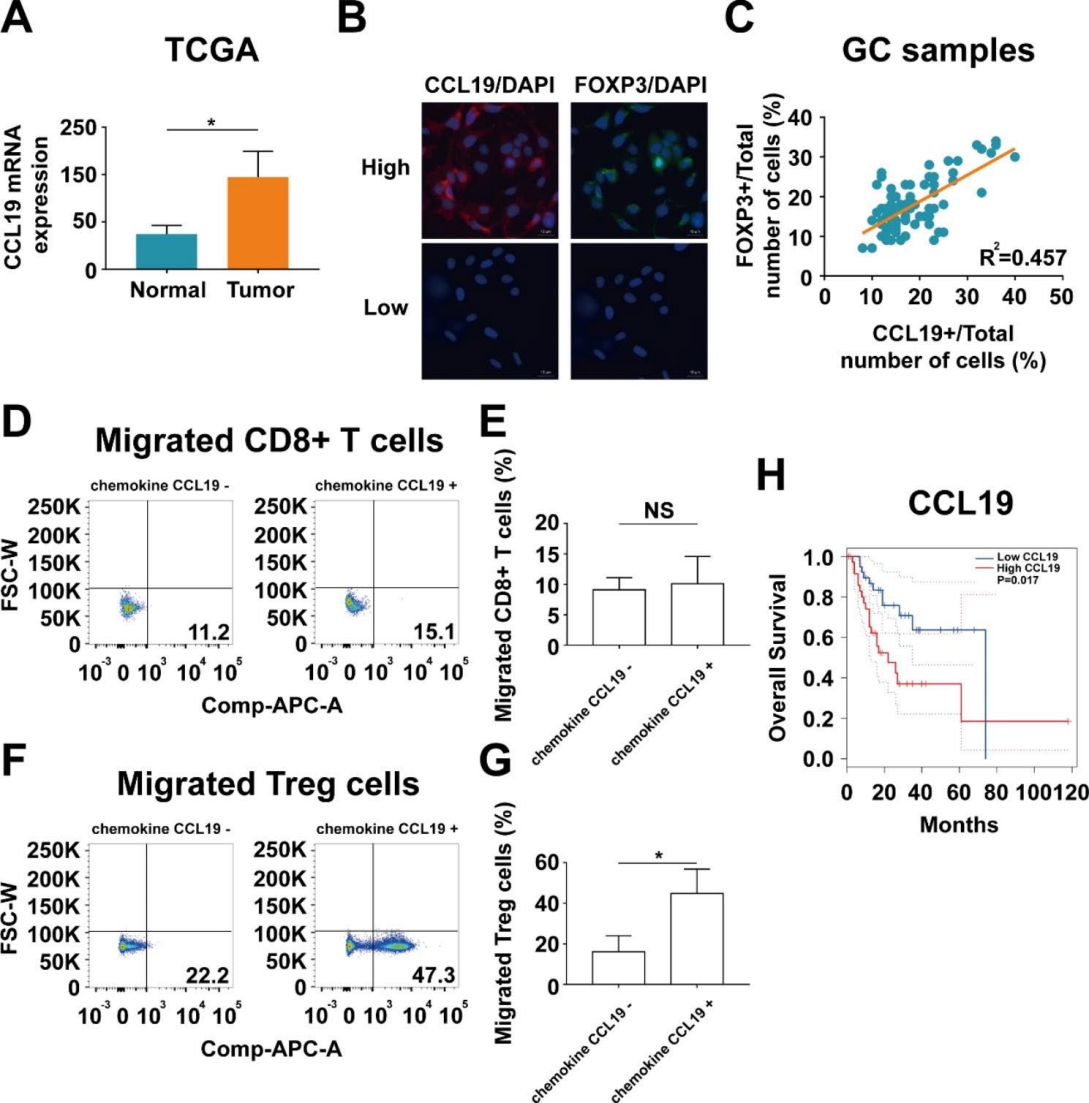
Chemokine CCL19 has strong chemotactic activity on Treg cells. (**A**) Relative CCL19 mRNA expression in 32 pairs of cancerous and noncancerous tissues from gastric cancer patients in TCGA database. (**B-C**) Immunofluorescence staining showed that the percentage of CCL19 + cells were positively correlated with the percentage of FOXP3 + cells in gastric cancer tissues. (**D-E**) Transwell experiment showed that the number of migrated CD8 + T cells was slightly increased in supernatant containing chemokine CCL19. (**F-G**) Transwell experiment showed that the number of migrated Treg cells was remarkably increased in supernatant containing chemokine CCL19. (**H**) Survival analysis of gastric cancer patients in TCGA database demonstrated that the patients with high CCL19 expression had worse overall survival than those with low CCL19 expression. Values are mean ± s.d., *P < 0.05 (Student’s t-test)

Comparing gene expression in 32 pairs of cancerous and noncancerous tissues from gastric cancer patients in TCGA database showed that CCL19 expression was significantly upregulated in gastric cancer samples compared with that in matched normal samples (Fig. 4A). We next detected CCL19 and FOXP3 in gastric cancer tissues by immunofluorescence staining. The results showed that the percentage of CCL19 + cells were positively correlated with the percentage of FOXP3 + cells in tumor tissues (Fig. 4B C). To determine the influence of chemokine CCL19 on Treg cells and CD8 + T cells migratory capacity, Treg cells or CD8 + T cells were co-cultured with BGC-823 cell lines adding chemokine CCL19 or not. The results showed that the number of migrated Treg cells was remarkably increased in supernatant containing chemokine CCL19, whereas the number of migrated CD8 + T cells was slightly increased in supernatant containing chemokine CCL19 (Fig. 4D and G). Survival analysis of gastric cancer patients in TCGA database demonstrated that the patients with high CCL19 expression had worse overall survival than those with low CCL19 expression (Fig. 4H), suggesting that CCL19 indicates poor prognosis in gastric cancer.

### CCR7 expression is elevated in gastric cancer samples compared with that in normal samples

**Fig. 5:**
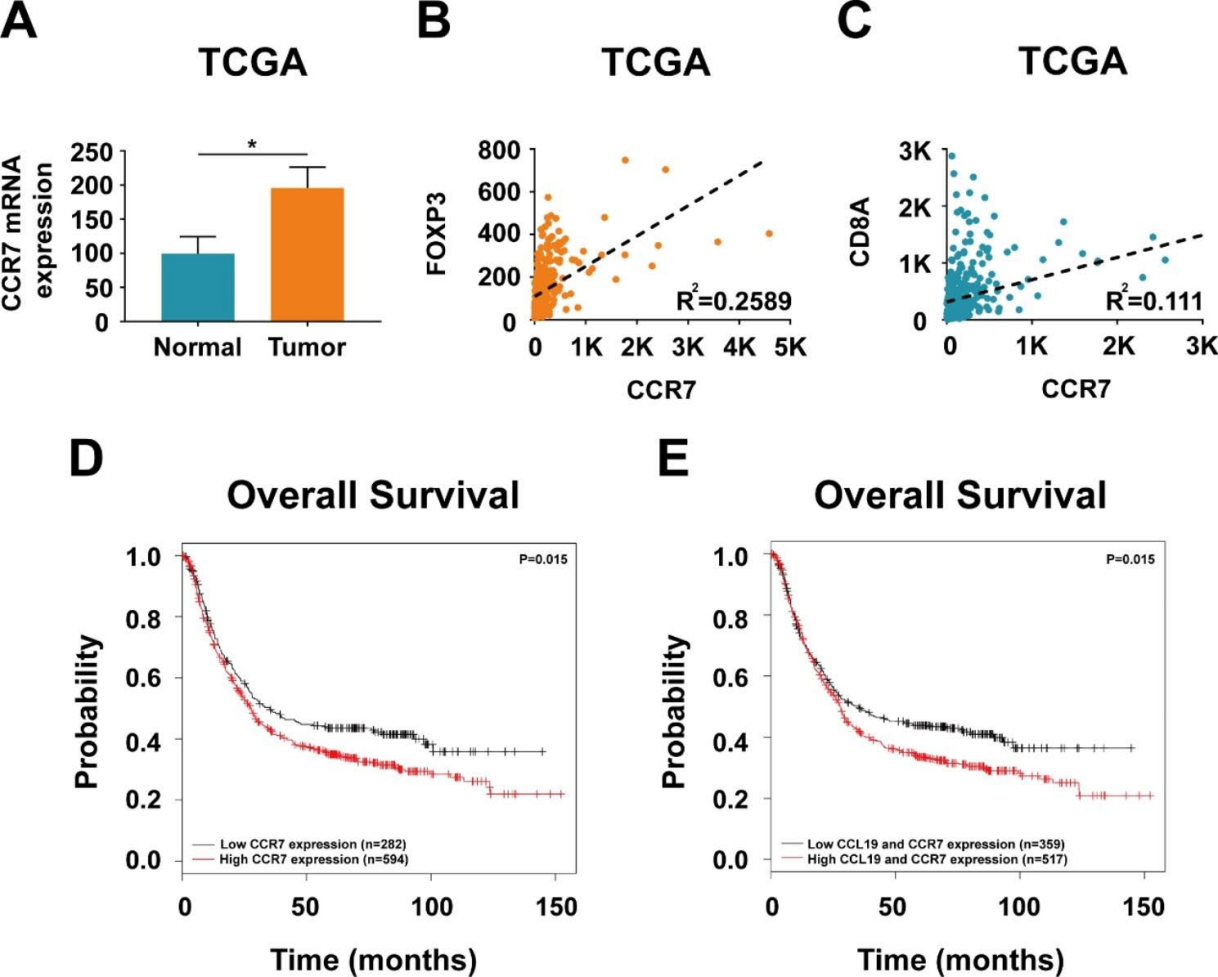
CCR7 expression is elevated and predicts poor prognosis in gastric cancer. (**A**) Relative CCR7 mRNA expression in 32 pairs of cancerous and noncancerous tissues from gastric cancer patients in TCGA database. (**B-C**) Pearson’s correlation coefficient between CCR7 and FOXP3, CCR7 and CD8A in TCGA database. (**D**) KMplotter database demonstrated that patients with high CCR7 expression had worse overall survival than those with low CCR7 expression. (**E**) KMplotter database demonstrated that patients with high CCL19 and CCR7 expression had worse overall survival than those with low CCL19 and CCR7 expression. Values are mean ± s.d., *P < 0.05 (Student’s t-test)

Then we analysed CCR7, the receptor of chemokine CCL19, in 32 pairs of cancerous and noncancerous tissues from gastric cancer patients in TCGA database. CCR7 expression was found to be significantly increased in tumor samples compared with that in matched normal samples (Fig. 5A). CCR7 showed strong correlation with FOXP3 expression and weak correlation with CD8A expression (Fig. 5B C). Analysis of 876 gastric cancer patients in KMplotter database demonstrated that patients with high CCR7 expression had worse overall survival than those with low CCR7 expression (Fig. 5D). The results also showed that patients with high CCL19 and CCR7 expression had worse overall survival (Fig. 5E), suggesting that CCL19/CCR7 indicates poor prognosis in gastric cancer.

## Discussion

Despite the rapid development in medical technology, gastric cancer is still a major public health problem, a serious threat to human life and a huge drain on social resources. Gastric cancer is common in Asia, and east and south-central Europe [24, 25]. Gastric cancer in the early stage can achieve complete remission through surgeries or endoscopic treatments [26]. Nonetheless, most gastric cancer patients are already in the progressive stage when diagnosed, leading to difficulties in treatment. Five-year survival rate for gastric cancer patients in the late stages had poor prognosis [27, 28]. Drugs targeting human epidermal growth factor receptor-2 (HER-2), epidermal growth factor receptor (EGFR), vascular endothelial growth factor (VEGF) have provided gastric cancer patients with better survival rates [29, 30]. Immune checkpoint inhibitors have successfully promoted long-term anti-tumor immune responses in patients with advanced cancer, such as metastatic melanoma [31–33]. However, there remains a large proportion of patients who do not benefit from checkpoint-blockade immunotherapy. There is a pressing need to explore novel and effectual molecular targets in order to ameliorate the poor prognosis of gastric cancer.

In our study, we discovered positive correlation between Treg cells and CD8 + T cells in gastric cancer tissues. Treg cell expression was significantly upregulated in tumor samples compared with that in matched normal samples and patients with high FOXP3 expression had worse overall survival than those with low FOXP3 expression, suggesting that Tregs cells indicate poor prognosis in gastric cancer. Then we analysed the relationship between chemokines and Treg cells or CD8 + T cells in gastric cancer. We found that only chemokine CCL19 had strong correlation with FOXP3 and weak correlation with CD8A. We also found that chemokine CCL19 had strong impact on the migratory capacity of Treg cells but weak impact on the migratory capacity of CD8 + T cells. Both CCL19 and CCR7 expression were significantly upregulated in gastric cancer samples compared with that in matched normal samples. High CCR7 expression had been reported to be associated with poor overall survival in gastric cancer [34]. Our research came to a similar conclusion that CCL19/CCR7 indicated poor prognosis in gastric cancer. However, our article focused more on the chemokine CCL19 and a series of analyses and experiments were carried out. The results suggested that blocking CCL19/CCR7 could inhibit Treg cell infiltration, but not affect CD8 + T cell infiltration. Therefore, both CCL19 and CCR7 may be the potential novel therapeutic targets in gastric cancer.

## Conclusions

Currently gastric cancer is still lacking for efficient targeted therapies. Although radical surgeries for gastric cancer in the early stages can cure a proportion of patients, those with advanced gastric cancer are still facing great challenges [35]. Effective biological target therapies are considered to improve survival time and prolong recurrence. Further studies should be conducted in order to obtain new therapeutic targets against gastric cancer.

## Data Availability

The data that support the findings of this study are available from the corresponding author.

## Abbreviations

BSA: Bovine serum albumin
CTLA-4: Cytotoxic T-lymphocyte antigen 4
DAB: Diaminobenzidine
EGFR: Epidermal growth factor receptor
FBS: Fetal bovine serum
FDA: Food and Drug Administration
HER-2: Human epidermal growth factor receptor-2
IHC: Immunohistochemical
OS: Overall survival
PBMCs: Peripheral blood mononuclear cells
PD-1: Programmed cell death protein 1
TCGA: The Cancer Genome Atlas
TGF-β: Transforming growth factor-β
TME: Tumor microenvironment
VEGF: Vascular endothelial growth factor
